# High-resolution assessment of the carrying capacity and utilization intensity in mountain rangelands with remote sensing and field data

**DOI:** 10.1016/j.heliyon.2023.e21583

**Published:** 2023-10-31

**Authors:** Harald Zandler, Kim André Vanselow, Sorosh Poya Faryabi, Ali Madad Rajabi, Stephane Ostrowski

**Affiliations:** a Department of Geography and Regionals Science, University of Graz, Heinrichstr. 36, 8010 Graz, Austria; b Institute of Geography, Friedrich-Alexander-Universität Erlangen-Nürnberg, Wetterkreuz 15, 91058 Erlangen,Germany; c Wildlife Conservation Society Afghanistan, Kabul, Afghanistan; d Wildlife Conservation Society (WCS), Bronx, NY, USA

**Keywords:** Dryland remote sensing, Grasslands, Grazing potential, Rangeland conservation, Vegetation modeling, Vegetation monitoring

## Abstract

Dry rangelands provide resources for half of the world's livestock, but degradation due to overgrazing is a major threat to system sustainability. Existing carrying capacity assessments are limited by low spatiotemporal resolution and high generalization, which hampers applied ecological management decisions. This paper provides an example for deriving the carrying capacity and utilization levels for cold drylands at a new level of detail by including major parts of the transhumance system. We combined field data on vegetation biomass and communities, forage quality, productivity, livestock species and quantities, grazing areas and their spatiotemporal variations with Sentinel-2 and MODIS snow cover satellite imagery to develop maps of forage requirements and availability. These products were used to calculate carrying capacity and grazing potential in the Pamir-Hindukush Mountains. Results showed high spatial variability of utilization rates between 5% and 77%. About 30% of the area showed unsustainable grazing above the carrying capacity. Utilization rates displayed strong spatial differences with unsustainable grazing in winter pastures and at lower elevations, and low rates at higher altitudes. The forage requirements of wild herbivores (ungulates and marmots) were estimated to be negligible compared to livestock, with one tenth of the biomass consumption and no increase in unsustainably grazed pastures due to the wider distribution of animals. The assessment was sensitive to model parameterization of forage requirements and demand, whereby conservative scenarios, i.e. lower fodder availability or higher fodder requirements of livestock due to climate and altitude effects, increased the area with unsustainable grazing practices to 50%. The presented approach enables an in-depth evaluation of the carrying capacity and corresponding management actions. It includes new variables relevant for transhumance systems, such as the combination of forage quantity and quality or accessibility restrictions due to snow, and shows utilization patterns at high spatial resolutions. Regional maps allow the identification of unsustainable utilization areas, such as winter pastures in this study.

## 1 Introduction

Arid and semi-arid rangelands cover over one third of the global land area and provide the basis for half of the world's livestock [1,2]. These regions offer manifold ecosystem services, including soil fertility, pollination or carbon sequestration, and harbor a high diversity of flora and fauna [3]. While grazing is critical for grassland ecosystem functioning, and extensive grazing by livestock or wild herbivores may foster biodiversity [4], overgrazing is considered to be the main driver for degradation of rangelands [1,5]. Additionally, climate is a dominant factor for ecosystem variability, which increases challenges due to accelerating environmental change and the need for sustainable management of rangelands [2,6,7]. Sustainable management requires accurate monitoring and detailed knowledge about the carrying capacity and utilization intensity [8]. However, the assessment of the grazing potential is challenging, as it involves a large number of parameters and a detailed database, such as the spatial vegetation distribution, respective forage quality, i.e. the nutritive energy content of different vegetation communities, biomass quantities, spatiotemporal variation of grazing areas, herbivore numbers and group structure [9]. Therefore, sound carrying capacity evaluations are missing in many regions [10]. Additionally, the large climate variability of drylands introduces high complexity and stochasticity in carrying capacity assessments. Recent studies linking satellite data and grazing utilization metrics showed that remote sensing approaches combined with field data offer promising tools for rangeland management and monitoring [8,11,12]. Furthermore, remote sensing is an essential and attractive tool to provide spatially and temporally resolved information for the peripheral location of rangelands [13,14].

Currently, a number of approaches exist to assess the grazing potential or carrying capacity with remote sensing methods to support ecological management approaches [13]. The majority of recent studies used the MODIS Net Primary Production (NPP) product [15] to compare productivity with livestock numbers [10,[16], [17], [18]]. Other studies used normalized difference vegetation index (NDVI) of medium resolution platforms, such as MODIS, to derive fodder quantities or a combination of medium resolution sensors with higher resolution data [[19], [20], [21]]. Carrying capacity assessments were also derived from biomass models or allometric models using higher resolution sensors such as SPOT5 or Sentinel-2 [[22], [23]]. Finally, some methods included remotely sensed vegetation classifications with subsequent class based allocation of forage values to derive regional carrying capacity [12,24]. However, the presented studies are connected to some uncertainties, as they do not include all relevant parameters, as outlined by Vanselow et al. [9], for accurate grazing potential assessment. Frequently, the respective approaches do not consider forage quality or they are characterized by relatively low spatial resolution of over 30 m, which is too coarse to cover the heterogeneity of arid rangelands [25,26]. Commonly applied methods based on MODIS NPP exclude sparsely vegetated areas [15], rendering respective techniques not feasible for most areas in arid or semi-arid rangelands. Finally, the spatiotemporal variability of grazing in livestock transhumance systems, with grazing areas changing with the seasons, is not included in most studies. Therefore, existing approaches are connected to considerable uncertainties and improved methods for accurate carrying capacity assessments are required to allow for sustainable rangeland management in drylands [20,25].

Hence, this study aims to provide a comprehensive, in-depth example for determining the carrying capacity in a cold dryland region using state-of-the-art remote sensing methods and ecological field data. To factor in all relevant aspects, we develop validated maps of forage quantities and quality, and compare them to spatiotemporal livestock forage requirements calculated from detailed livestock metrics to assess grazing potential and utilization patterns. Finally, we present key factors that influence the analysis and discuss involved uncertainties to guide future assessments in arid rangeland ecosystems.

## 2 Materials and methods

### 2.1 Research area

**Fig. 1:**
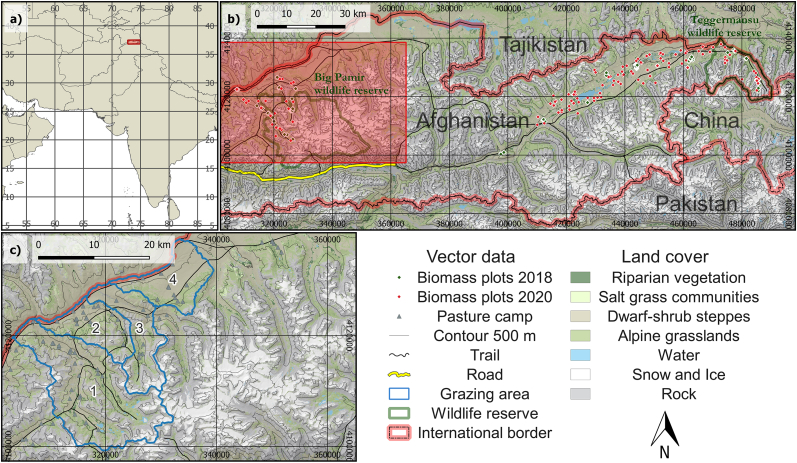
Location (a) and overview (b) of the research area, field plots, land cover; and detail of selected grazing areas (c, 1: Shikarga, 2: Manjulak, 3: Alisu, 4: Jirmasirt) for the analysis. DEM: NASA JPL [34], borders: GADM [35], land cover classification: Zandler et al. [14].

Our research focuses on a region that is exemplary for dry rangelands, with livestock grazing as the main economic activity and relatively low vegetation cover, the Eastern Wakhan of Afghanistan (Fig. 1a–c). This high-mountain area, with elevations between 3000 m and 6000 m, is the source of the Amu Darya watershed with supra-regional importance for millions of people as one of the continent's main “water towers” [27,28]. The climate is cold and dry, with yearly valley precipitation of around 100–200 mm, and annual mean temperatures around – 1 °C and – 6 °C [29,30]. Regional ecosystems harbor about 20% of endemic plant species, and an important population of large wild herbivores including the Marco Polo sheep (*Ovis ammon polii*), urial (*Ovis vignei*) and the Siberian ibex (*Capra sibirica*) [[31], [32], [33]]. Other rare species include the snow leopard (*Panthera uncia*) and the large-billed reed warbler (*Acrocephalus orinus*). The vegetation shows large heterogeneity due to varying water conditions, with low vegetation cover in steppe communities and medium cover in riparian or alpine grasslands [14]. The whole area, including high altitude pastures, is utilized for livestock grazing by domestic yaks, sheep, goats, cattle and, a few Bactrian camels. The high ecological value of the region led to the establishment of the Wakhan national park in 2014, including the Big Pamir and Teggermansu wildlife reserves (Fig. 1b). However, the impacts of projected infrastructure development, climate change and livestock grazing make remote sensing based monitoring and the assessment of the carrying capacity essential prerequisites for ecological management in the region [14].

### 2.2 General approach

Numerous definitions of the term “carrying capacity” exist [25]. In this study, we apply it to populations of livestock and consider the “carrying capacity” as the sustainable stocking rate of an ecosystem, while “grazing potential” refers to the maximum amount of forage available to all meso-mammalian herbivores, and “utilization intensity” is the percentage amount of available forage used by livestock in a specific grazing area. Respective variables allow for a sound evaluation of the sustainability of ecosystem use.

**Fig. 2:**
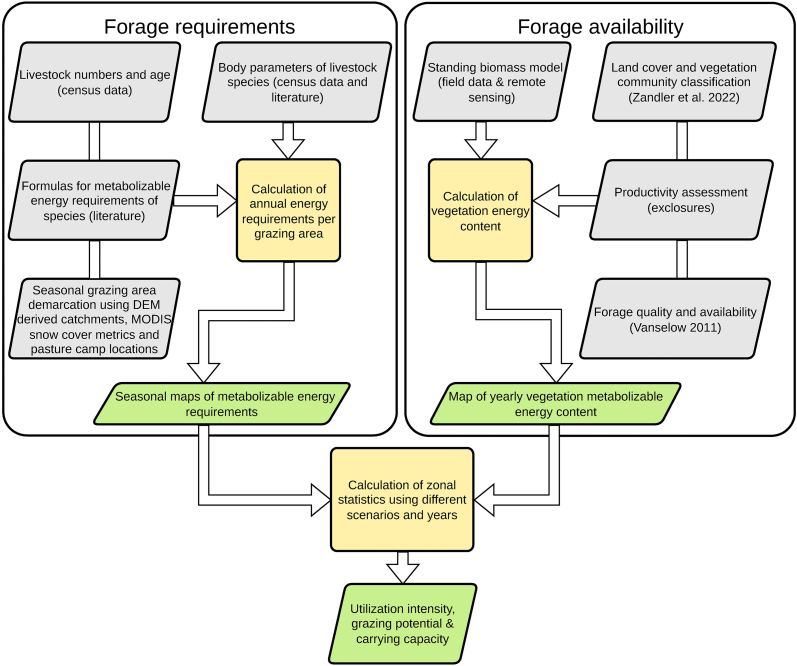
Methodology for the assessment of the carrying capacity, grazing potential and utilization intensity.

An accurate assessment of the carrying capacity requires information on livestock forage requirements and spatiotemporal forage availability [9]. These two main parts involve a large number of parameters (Fig. 2).

Forage availability, characterized by the Metabolizable Energy Content (MEC) of the vegetation, is based on biomass amounts and the forage quality, i.e. the nutrients and energy of the biomass, which depends on the species composition, and is spatially reflected by the distribution of vegetation communities. We use field data in combination with remote sensing modeling to derive and combine these parameters for a spatiotemporal map of MEC. Forage quality was assigned using a land cover classification with known nutritive properties per class, and biomass quantities per pixel were derived from a biomass model. Respective models were trained and evaluated by field plots of biomass and land cover in combination with exclosures (Section 2.7). Forage availability is therefore provided at the pixel level and a spatial resolution of 10 m. Forage requirements, represented by Metabolizable Energy Requirements (MER), are calculated based on the herd size and composition, which were covered by livestock census surveys. The livestock census was performed in all single pasture camps, but livestock grazing is managed using informal grazing areas with defined borders [12] and livestock is distributed within these units. Therefore, respective numbers of the derived MER are assigned to grazing areas for a spatial allocation of the data. Grazing areas vary temporally according to different seasons (spring, summer and winter pastures) and accessibility (snow). Finally, we aggregate data of MEC to the same levels as MER (grazing areas) to compare spatially resolved results and to derive the yearly carrying capacity, utilization intensity and grazing potential of the region.

### 2.3 Metabolizable Energy Requirements and livestock census

Various approaches to calculate MER of herbivores exist and most depend on the species, sex, nutritional status, weight and age of the animals [9,[36], [37], [38]]. However, the majority of validated measurements for livestock were empirically derived in high-intensity farming systems, low altitudes, stall-fed livestock or in temperate regions with concentrate or high energy diets. Therefore, we applied the allometric prediction for field metabolic rates for free-ranging mammals as given by Nagy et al. [39].

$$\left(1\right)ME=4.82*BM^{0.734}/1000$$

Thereby, ME is metabolizable energy expressed in megajoules (MJ) per day and BM is body mass (g) of the animals. For the latter, we used data for the end of each season to account for ME used for fat storage, in addition to oxidative metabolism in spring and summer, and more oxidized energy loss during winter leading to lower weight in spring. With this approach, gains and losses of body weights are included in the calculation. We derived seasonal MER sums using the respective ME formula in combination with livestock body mass and livestock numbers per seasonal grazing area from the livestock census (section 2.4), and by finally multiplying this result with the number of days per season.

However, several effects may additionally influence the energy demand of animals, such as high-altitude, low temperatures, rugged terrain, sparsely covered mountainous pasture, snow, animal pregnancy and lactation [[40], [41], [42], [43], [44]]. Although existing research indicates higher metabolic energy requirements in high mountain regions for livestock without a long evolutionary history in mountain regions, quantitative figures in literature vary according to species and the specific environmental situation [41,42,45].

To account for respective uncertainties, we additionally calculate a scenario with 30% increased MER for all species except yak, because their metabolism is likely to be evolutionarily adapted to the studied environment [45]. We used a 30% increase following to results presented in Degen and Young (2002) [42] and the mean annual temperature difference between valleys and high plateaus of around 10 °C outlined in Zandler et al. [46]. However, both temperature and altitude effects on metabolism showed strong variations in the mentioned literature, so different scenarios may be tested.

### 2.4 Livestock census

The livestock parameters for the formulas were derived from livestock census data available for the Western Big Pamir area for the years 2006 and 2010–2020 [47]. Winter surveys were conducted in 2015, 2016 and 2017. Thereby, winter camps were visited in early spring, and summer camps in high summer or early autumn. Groups of two surveyors performed counts with hand counters in the pasture camps. Older (>1 year) and younger animals (<1 year) were recorded separately. Additional surveys were performed for sheep in 2016 and 2017 (n = 58). Thereby, each animal was weighted in spring and fall to assess total body mass and daily gain of body mass in the different age classes. For other species, no such data is available. Therefore, we derived BM values for yak, goats, cattle and camels in different age groups from research results in comparable environments [44,[48], [49], [50], [51], [52], [53]] and personal communications with regional slaughterhouses (e.g. Khorugh, Tajikistan). For years without winter censuses, we derived livestock numbers for this season by using the average winter stocking rates. Respective winter stocking rates were calculated as the average ratio between summer stocking rates and winter stocking rates during the three years with available winter livestock count results (2015, 2016, 2017). For cattle, which graze only during the high-summer months, counts were only available for one year and so identical numbers for all years were assumed. Winter numbers were attributed to the previous growing season, i.e., animals of the early spring census 2017 were counted for the 2016 growing season.

### 2.5 Spatiotemporal grazing area demarcation

To derive grazing areas (Fig. 1c), we merged catchment areas with GPS data of pastures camps, field maps of pastures and qualitative information from field interviews. The catchments were derived using a digital elevation model (DEM) [34] and delineation algorithms of the QGIS software [54]. To consider spatiotemporal variations of transhumance systems, we repeated the analysis using different areas and seasons dependent on local habits. Thereby, livestock numbers were attributed to grazing areas, but livestock grazing was restricted depending on the season.

The vegetation period starts in spring and early-summer (May–June), whereby herders still reside in winter or spring camps. Snow still covers high altitudes during this period, so grazing is limited to lower elevations. Therefore, we used the MODIS MOD10A1 V6 product [55] with a resolution of 500 m to derive seasonal maps of Fractional Snow Cover (FSC) [30]. We compared respective maps to seasonal pasture camp locations and field information on grazing areas to derive a snow cover threshold under which grazing occurs. For the May–June season, grazing occurred in all areas below 35% of FSC. In high-summer and the beginning of autumn (July–September), sheep and goats are also able to graze at higher altitudes, but usually grazing is restricted to areas below 4500 m a.s.l. for these species, so we used this elevation as a threshold. Yak and cattle also graze the upper regions during this period. Thereby, grazing concentrates on high altitudes [56], so no upper elevation restriction was considered, but winter pastures were excluded. Winter grazing (October–April) was finally limited to grazing areas with winter camps and livestock occurrence based on the winter census. Again, snow is a main limiting factor for useable pasture area during this period, but grazing still occurs up to a considerable degree of snow cover. Based on camp locations and qualitative field data, we restricted grazing to areas with a mean winter FSC below 78%. Livestock in pasture camps solely relies on grazing and no stored dry fodder is consumed.

### 2.6 Wild herbivores

In addition to livestock, wild herbivores such as Marco Polo sheep, Siberian ibex and long-tailed marmots (*Marmota caudata*) contribute to grazing pressure in the region (cf. [10]) Although the focus of this paper is livestock utilization, we also include a scenario with consumption estimates of the aforementioned wild herbivores in the presented assessment. Marmot numbers were estimated using regional observations [57], resulting in densities of approximately 16.3 individuals per km²[], which is comparable to published values of other regions [58,59]. Body masses in different seasons were derived from literature values [59,60]. We considered all vegetated areas as potential marmot habitat for the assessment and took hibernation into account. For Marco Polo sheep and Siberian ibex, we derived regional numbers, weights and seasonal weight variations from literature values [56,[61], [62], [63], [64]]. For wild herbivores, we did not consider any grazing area restrictions.

### 2.7 Spatiotemporal forage availability

Spatiotemporal MEC of vegetation depends on total biomass amounts per year in kg dry matter (DM) and the energy content of plants per kg DM. Therefore, we combined standing biomass maps with productivity estimates, and vegetation communities with laboratory analysis of forage quality. Finally, we considered wasted biomass amounts and constraints for forage quantities that can practically be consumed by herbivores, leaving only a fraction of this amount for ingestion by the animal [44,65]. According to existing research, the actual forage amount consumable by livestock is only 50% for dwarf-shrub deserts and steppes due to the abundance of woody biomass and hard shrub cushions, and 80% for all other vegetation communities [44,66]. Respective fractions were used to calculate the final spatiotemporal MEC of the research area.

#### 2.7.1 Forage quality

We used the vegetation community classification from Zandler et al. [14] as the basis for creating forage quality maps. Respective classification, which was modeled with field, Sentinel-2 and DEM data using a random forest algorithm, showed good cross-validated overall accuracy of about 80% in different years and has a spatial resolution of 10 m. It includes all major vegetation communities, such as riparian grasslands, *Salix* sp. riparian communities, salt grass communities, dwarf-shrub deserts and steppes, alpine grasslands and other, largely non-vegetated land cover classes such as water surfaces, snow and ice, dark rocks and scree and bright rocks and scree [14]. We excluded all non-vegetated classes for subsequent analysis of forage availability.

To assign values of ME in MJ per kg DM to the vegetation classes, we utilized laboratory results from the study of Vanselow 2011 [44]. Data from this study refers to the Eastern Pamirs of Tajikistan, which neighbors (<70-km distance) the research area and is characterized by similar vegetation communities with identical dominant species [14]. ME of the plants was derived using different samples of the vegetation communities with subsequent laboratory analysis of neutral detergent fibre, acid detergent fibre, acid detergent lignin, nitrogen (as a proxy for crude protein), crude fat, and crude ash. Total digestible nutrients were calculated according to Weiss et al. [67] and Van Soest [68]. Finally, respective values were converted to ME [69] and averaged to appropriate vegetation communities. Thereby, we assigned forage quality values of 9.4 MJ/kg DM to riparian grasslands and Salix riparian communities, 9.1 MJ/kg DM to salt grass communities and alpine meadows, and 6.3 MJ/kg DM to dwarf-shrub deserts and steppes. Existing research shows lower forage quality in winter compared to summer, but this ratio strongly varies and in the research area, only small differences were measured [44,70]. Therefore, no reduction was considered for winter energy amounts. Final ME values of different vegetation communities are within the range of results from other studies in rangeland ecosystems [[71], [72], [73]], but the large variation in values also shows the requirement for community adapted forage quality values.

#### 2.7.2 Standing biomass model

We mapped standing biomass in the field during two different campaigns in 2018 and 2020. The 2020 survey followed a random sampling design with stratification using the vegetation classification [14], and a 5 km buffer around the 2016 expedition route that covered the grazing areas and wildlife reserves. Thereby, 50 plots were randomly placed within dwarf shrub deserts and steppes, 30 in each of the classes riparian and alpine grasslands, and 15 in salt grass communities to reflect the relative abundance of the classes. At each plot, a square of 4 m side length was established according to recommendations in Mueller-Dombois & Ellenberg (1974) [74], with subsequent clipping and weighting of all non-woody vegetation (fresh and air dry). After quality control, standardization and conversion to kg DM/100 m^2^, 121 plots were left for the final analysis. This dataset was used for creating and validating the standing biomass model. The 2018 survey followed a different sampling design, as it was originally designed for a resurvey of permanent monitoring plots in the national park (cf. [14]). Biomass clipping was implemented on 1 m^2^ plots, weighted and converted to kg dry biomass/100 m^2^ using a linear fresh-dry model (R^2^ = 0.89, n = 67), which was established with independent field samples. Respective samples for the establishment of the fresh-dry model were additionally collected during several survey campaigns between July and September from 2016 to 2020. Residuals showed small variations and approximately equal variances indicating the applicability of the model. Variations in the fresh-dry ratio of the samples were generally small. We used the values of the 2018 survey sites (n = 111), situated at different locations compared to the 2020 survey, to validate the temporal and spatial transferability of the 2020 model, but not for training of remote sensing models.

To create the spatial biomass model, we converted Level-1C Sentinel-2 data [75] to surface reflectance using the sen2cor software [76]. The original product already includes geometric correction with geolocation errors below 6 m [77], including mountain areas, which is considered sufficient for the presented approach. We resampled 20 m bands to 10 m using a nearest-neighbor algorithm, and extracted spectral information for the available biomass plots. Vegetation indices for the model were selected based on previous modeling results (Table 1), which indicate that soil adjusted indices in the red and near-infrared domain [78], red-edge indices [79] and short-wave infrared indices are important for biomass modeling in dry rangelands of Central Asia [14].

**Table 1:** Selected vegetation indices for the biomass model. Wavelength definitions are presented in ESA (2020) [75]. The coefficients α and β corresponds to the slope and the intercept of the soil line from Zandler et al. [78].

Index	Formula	Reference
WDVI	Band 8-(α*Band 4)	[80]
WDVI SA	WDVI -(0.45*(Band 4-Band 3)/(Band 4+Band 3))	[81]
Ratio B8a/B3	Band 8a/Band 3	[82]
NDRE 1	(Band 8-Band 7)/(Band 8+Band 7)	[83]
NDRE 2	(Band 8-Band 8a)/(Band 8+Band 8a)	[83]
MTCI	(Band 6-Band 5)/(Band 5+Band 4)	[84]
SACRI	(α*(Band 8-Band 11-β))/(α*Band 8+Band 11-α*β)	[85]
MSACRI	5*(α*(Band 11-α*Band 12-β))/(α*Band 11+Band 12+α*β)	[85]

We selected the random forest algorithm to model biomass amounts [86], as multiple studies showed that the approach is still among the best performing methods using medium resolution remote sensing imagery [14], due to its robustness [87] and as it was successfully applied for vegetation quantification in similar environments [14,79,88]. The model was set to 500 trees with other parameters unchanged, as parameter tuning is expected to only marginally change results with this method [89]. To generate unbiased error estimates, we performed 100-repeated 10-fold spatial cross validation using the functionality of the R packages mlr and mlr3 [[90], [91], [92]], and calculated the cross-validated performance measures R^2^, RMSE (Root Mean Squared Error), RMSErel (relative RMSE), MAE (Mean Absolute Error), MAErel (relative MAE), BIAS and BIASrel [46]. With the derived model parameters, we calculated standing biomass using near cloudless scenes in July for the years 2016–2020. Finally, we repeated the calculation of performance metrics using the independent 2018 field data and the predicted values for this year.

#### 2.7.3 Biomass productivity

Various methods exist to measure aboveground net primary plant production, i.e. productivity, with different advantages and disadvantages [93]. We selected the exclosure plot method and clipping at the end of the vegetation period (September), as this is the most common and comparable approach [44]. To derive kg DM, all samples were dried to constant mass after clipping. Clipping was also performed at identical areas outside of the exclosure plots to derive the relationship between standing biomass and total productivity. This ratio was used to calculate annual biomass amounts from the standing biomass map. Therefore, 13 exclosure plots were constructed to cover different vegetation communities. Unfortunately, logistical constraints resulting from the changing political situation in Afghanistan prevented measurement of all plots in some years and several plots were destroyed by wildlife or human activities, leaving only four plots for derivation of productivity and the relationship to standing biomass in grazed areas.

To consider uncertainties connected to forage availability due to model errors and productivity variations, we calculated an additional conservative biomass scenario by lowering modeled biomass amounts by 50% of the mean MAE of both validation years. This value was selected arbitrarily to provide a pessimistic, but not extreme scenario.

### 2.8 Sustainable utilization

Several authors consider 50% as the maximum rate for sustainable utilization in grassland ecosystems [8,[94], [95], [96]]. However, utilization rates vary in different ecosystems, whereby lower utilization levels are required in dryer environments or with increased altitude [97]. Therefore, we considered a utilization level of 40% as sustainable in the dry, high-mountain rangelands of the research areas following guidelines from existing studies in approximately similar landscapes [97,98].

## 3 Results

### 3.1 Standing biomass model and productivity

The standing biomass model for the year 2020 resulted in a cross-validated R^2^ of 0.55, a RMSE of 2.23 kg DM/100 m^2^ and a MAE of 1.7 kg DM/100 m^2^ compared to a mean biomass of 3.27 kg DM/100 m^2^ and a standard deviation (SD) of 3.53 kg DM/100 m^2^, measured in the field (Table 2). Largely similar results were calculated for 2018. The validation dataset of 2018 also contained an outlier with unusually high biomass values. Removal of the respective plot would lead to reduced errors and a R^2^ of 0.69, RMSE of 1.63 kg DM/100 m^2^ and a MAE of 1.14 kg DM/100 m^2^ in 2018. Both years showed negligible model bias.

**Table 2:** Performance measures of the standing biomass model. Values for the 2020 model were calculated using spatial cross-validation (CV) and the 2018 data with independent field data.

	R2	RMSE (kg DM/100 m [2]))	RMSErel	MAE (kg DM/100 m [2]))	MAErel	BIAS (kg DM/100 m [2]))	BIASrel	Mean field biomass (kg DM/100 m [2]))	SD (kg DM/100 m [2]))
2020 (CV)	0.55	2.23	68.36	1.7	52.05	−0.09	−2.82	3.27	3.53
2018	0.55	2.44	86.61	1.31	46.47	0.17	5.98	2.82	3.61

Total yearly productivity estimates averaged for different vegetation communities generally showed the highest amounts in riparian communities, followed by alpine grasslands. Salt grass communities showed lower amounts, whereas dwarf-shrub deserts and steppes are characterized by the lowest yearly biomass productivity (Table 3). Temporally, biomass amounts showed different developments depending on the vegetation community with opposite developments of azonal riparian areas compared to zonal communities. Averaged over the whole area, the largest biomass amounts were available in 2017, followed by 2016, 2019, and 2018, and the lowest values in 2020.

**Table 3:** Total biomass productivity (kg DM/ha) averaged over different vegetation communities and years.

	2016	2017	2018	2019	2020
Riparian grasslands	1609	1745	1762	1866	1663
Salix riparian communities	1900	1964	1994	2030	2023
Salt grass communities	679	715	636	624	553
Dwarf-shrub deserts and steppes	535	556	420	435	357
Alpine grasslands	941	1005	881	887	787

### 3.2 Metabolizable energy amounts

**Fig. 3:**
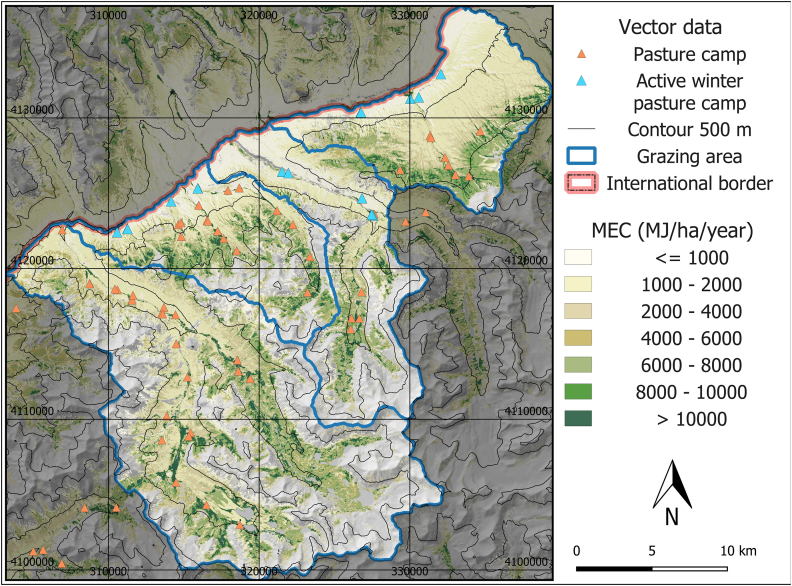
Map of mean modeled Metabolizable Energy Content (MEC) 2016–2020 in MJ per ha per year for the detailed analysis. Active winter pasture camps are camps with present livestock during the winter surveys. Pasture camps are all other camp locations, including currently abandoned winter camps.

The averaged modeled yearly MEC amounts (2016–2020) available to herbivores were 12967 MJ/ha in riparian grasslands, 14866 MJ/ha in Salix riparian communities, 4682 MJ/ha in salt grass communities, 1428 MJ/ha in dwarf-shrub steppes and 6573 MJ/ha in alpine grasslands. Spatially, a clear gradient depending on altitude was visible, with lower amounts in lower elevations and higher amounts in high altitudes (Fig. 3).

### 3.3 Livestock numbers and energy requirements

Livestock numbers in summer showed the highest values in 2013 (Table 4), with relatively lower numbers during the satellite analysis period from 2016 to 2020. In the latter period, the highest livestock numbers were surveyed in 2020 in summer and 2016 in winter, whereas the lowest livestock numbers were registered in 2019 in both periods.

**Table 4:** Livestock numbers (summer/winter) of all age classes during different years. Total number is summarized as small livestock units (SLU), reflecting the headcount of small livestock (sheep and goats) plus large livestock times three [12].

Species	2006	2010	2011	2012	2013	2014	2015	2016	2017	2018	2019	2020
Goat	1059/205	3663/451	4054/438	3886/413	4982/722	5490/350	5151/504	4547/770	4555/770	5349/917	4801/760	5596/875
Sheep	6525/1017	8101/1070	7344/938	8046/1023	11017/1404	10093/3177	10306/3966	9187/4295	9033/4295	9321/2070	8110/1871	9576/2214
Yak	683/106	905/114	949/102	915/86	1081/118	936/145	966/233	907/205	978/205	764/138	726/123	849/146
Cattle	982/0	982/0	982/0	982/0	982/0	982/0	982/0	982/0	982/0	982/0	982/0	982/0
Camel	NA	NA	NA	NA	NA	NA	NA	30/0	30/0	17/0	31/0	22/0
**Total sum (SLU)**	12579/1540	17425/1863	17191/1682	17623/1694	22188/2480	21337/3962	21301/5169	19491/5680	19558/5680	19959/3401	18128/3000	20731/3527

Daily MER of species varied between the seasons, and according to their respective body masses was highest in yaks, followed by cattle, sheep and goats (Table 5). Estimated annual energy requirements for livestock kept on pastures year-round were 13789 MJ for adult yak, 4207 MJ for adult sheep and 3798 MJ for adult goats.

**Table 5:** Daily energy requirements in MJ/day per species per season.

Species	Early summer (>1 year/<1 year)	High summer (>1 year/<1 year)	Winter (>1 year/<1 year)
Goat	10.87/8.25	13.67/11.11	8.86/6.14
Sheep	11.96/9.79	14.68/12.51	10.03/7.83
Yak	38.42/17.28	42.80/20.6	35.41/13.75
Cattle	NA	40.18/NA	NA
Camel	NA	77.49/NA	NA

### 3.4 Grazing potential, carrying capacity and utilization intensity

On average one ha of pasture of riparian grasslands would provide grazing potential for one year to a maximum of about one yak or three sheep or about 3.5 goats, whereas the carrying capacity would be about 0.4 yak or 1.2 sheep or 1.4 goats (Table 6). The comparison of different vegetation communities shows the highest grazing potential for *Salix* riparian communities, followed by riparian grasslands, alpine grasslands, salt grass communities and dwarf-shrub steppes with the lowest potential.

**Table 6:** **Annual livestock** grazing potential and carrying capacity averaged over all years (2016–2020) for different vegetation communities in the research area.

	Annual grazing potential (animals/ha)			Annual livestock carrying capacity (animals/ha)		
Vegetation Community	Yak	Sheep	Goat	Yak	Sheep	Goat
Riparian grasslands	0.94	3.08	3.41	0.38	1.23	1.37
Salix riparian communities	1.08	3.53	3.91	0.43	1.41	1.57
Salt grass communities	0.34	1.11	1.23	0.14	0.45	0.49
Dwarf-shrub steppes	0.10	0.34	0.38	0.04	0.14	0.15
Alpine grasslands	0.48	1.56	1.73	0.19	0.62	0.69

**Fig. 4:**
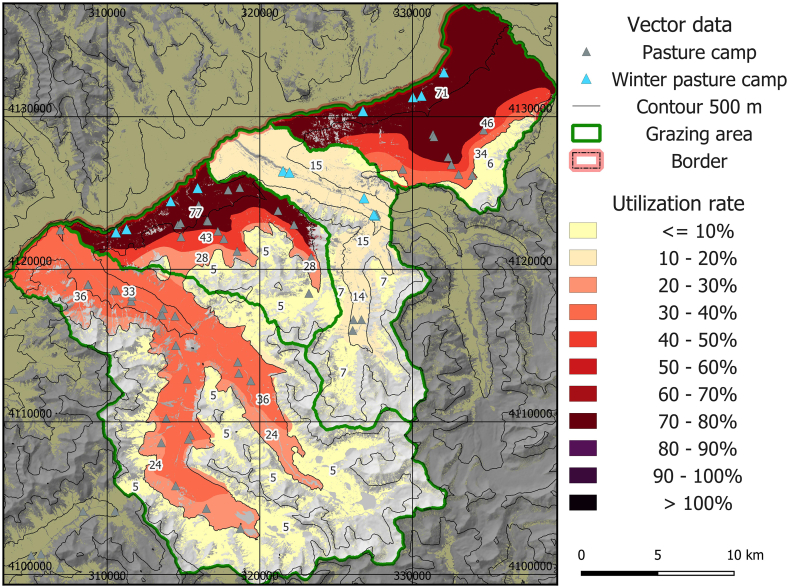
Map of mean livestock utilization rate 2016–2020 for the basic model.

The spatial assessment showed a large range of utilization levels, with grazing utilization ranging between 5% and 77%, and about 30% of the area showing unsustainable utilization rates in the basic model (Fig. 4). Generally, high altitudes and summer grazing areas showed low utilization levels and increased utilization was found at lower elevations and in grazing areas with high livestock numbers in winter. The grazing areas Manjulak (number 2 in Fig. 1c) and Jirmasirt (4 in Fig. 1c) were most affected by overutilization, whereas Shikarga (1 in Fig. 1c) was slightly below the 40% threshold of unsustainable utilization. Alisu (3 in Fig. 1c) showed sustainable livestock utilization levels according to the basic model.

To provide some information on seasonal differences, we also calculated utilization numbers without winter grazing. This would lower the utilization intensity on the lower part of Manjulak (2 in Fig. 1c) from 77% to 39%, and from 71% to 43% in Jirmasirt (4 in Fig. 1c). Considering the whole area, 22% showed unsustainable utilization rates without winter grazing. Respective areas were located within winter and early summer grazing areas.

**Fig. 5:**
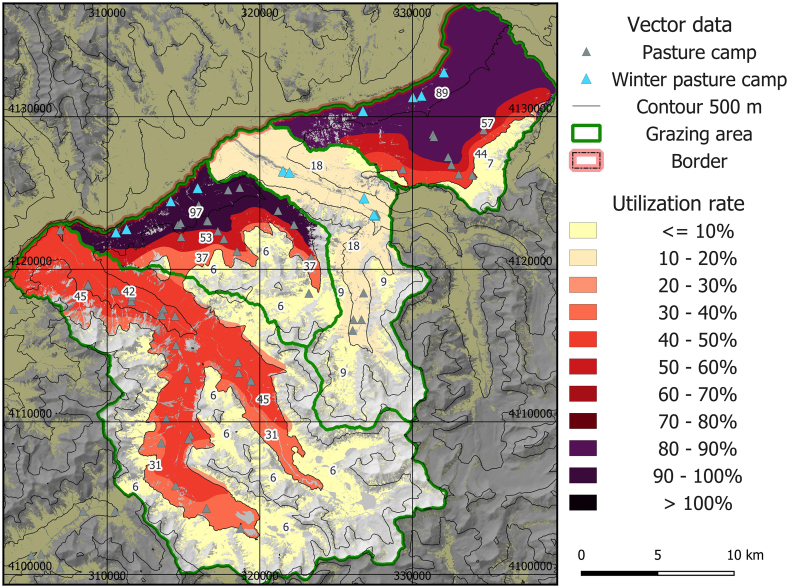
Map of mean livestock utilization rate 2016–2020 for the scenario with 30% increased metabolized energy requirement (MER) for all species except yak to account for high-altitude and low temperature effects.

Respective numbers were considerably higher using a potential scenario of increased energy demand of livestock due to high altitude and low temperatures (Fig. 5). Thereby, almost all available forage would be utilized in Manjulak (2 in Fig. 1c) and Jirmasirt (4 in Fig. 1c), and in Shikarga (1 in Fig. 1c), the sustainability threshold would be crossed. Alisu (3 in Fig. 1c) and high altitude regions still showed sustainable utilization levels. Fifty percent of the area is unsustainably grazed in this scenario.

**Fig. 6:**
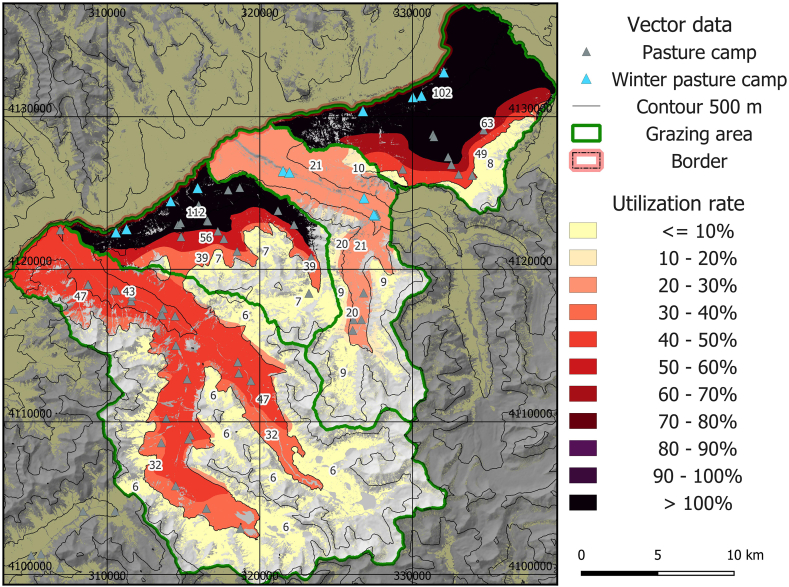
Map of mean utilization rate 2016–2020 for the conservative biomass scenario, which considers potential uncertainties of the biomass model by reducing biomass values by 50% of the MAE.

The conservative biomass scenario, which considers potential uncertainties of the biomass model by reducing biomass values by 50% of the MAE, showed the highest utilization rates of all models (Fig. 6). In two grazing areas, Manjulak (2 in Fig. 1c) and Jirmasirt (4 in Fig. 1c), the grazing potential is slightly exceeded. The utilization rate in Alisu (3 in Fig. 1c), mostly used in winter, is still far below the sustainability threshold with this model. Similar to the other scenario, about 50% of the area is above the utilization threshold for sustainable grazing practices.

The averaged utilization rate for the whole research area was 33% (2016–2020), with the highest value of 38% in 2020, followed by 2016 with 33%, 2018 and 2017 with 32 %, and 2019 with 28%.

The wild herbivore scenario resulted in an increase of around 0.7 percentage points in the mean utilization rate for marmots, and an increase of 0.8 percentage points for ungulates. Hence, the total increase in utilization rate is 1.5 percentage points averaged over the whole area, with a higher share up to 3.9 percentage points in two winter grazing areas. This scenario does not increase the area affected by unsustainable grazing practices compared to the basic scenario, but slightly increases the magnitude.

Compared to total energy requirements, MER of marmots corresponds to 5.6% of livestock requirements, and the requirements of ungulates (Marco Polo sheep and Ibex) sum up to 6.7% of the livestock demand, leading to 12.3% in total.

## 4 Discussion

### 4.1 General findings

To our knowledge, this is the first study that provides a high-resolution model of the livestock carrying capacity in a cold dryland region, including all key parameters and their spatiotemporal variations. The integration of snow variables for limiting the available pasture areas in cold dryland rangelands is an additional innovative feature in carrying capacity studies. Results show large spatial differences, with overutilization at lower elevations, in winter or early summer pastures, and relatively low usage in remote, high-elevation regions, a pattern that is in agreement with existing research in comparable environments [12,44,99]. The results show that in the winter season livestock numbers are currently maximal in terms of food competition with wild herbivores, with utilization rates approaching or surpassing 100% in the winter grazing areas in our scenarios, underpinning the importance of seasonal assessment approaches for ecologically based livestock, wildlife and conservation management [87]. The resulting spatial utilization patterns show good agreement with field surveys of overgrazing and degradation [100].

### 4.2 Wildlife utilization

Carrying capacity assessments from other regions indicated that in addition to livestock, wild herbivory may lead to the overutilization of pastures [10]. In the research area, however, wildlife related impacts on the utilization rate are comparably small. This is exemplified by the total energy requirements of wild herbivores that amount to about one tenth of livestock requirements in our study. Furthermore, due to the larger area grazed and the wider distribution of wildlife, utilization is less concentrated which leads to much lower impacts in terms of ecosystem sustainability. Therefore, no increase in the area affected by unsustainable grazing practices was modeled compared to the basic scenario of no wildlife grazing. However, areas already affected by overgrazing showed larger increases in the overutilization magnitude.

### 4.3 Sustainability and practical applications

In summary, these results show that although unsustainable grazing practices exist, particularly in most winter grazing areas and at lower elevations, the traditional herding system in the study site appears sustainable to a certain extent, but even so livestock stocking rates and available biomass were often not synchronized. Among other years, this is illustrated by the year 2020, with the highest livestock numbers in summer and the lowest biomass availability leading to the highest utilization rates. Timely carrying capacity forecasts based on climatological relationships could greatly support better management in this regard [30]. Potential approaches are biomass forecasts based on winter snow amounts or hydrological indicators of the previous year.

Our finding of sustainable aspects of traditional grazing practices was also reported in other regions [100]. Similarly, indigenous ecological knowledge could contribute to more adapted grazing practices in addition to ecological methods [101]. In the research area, several factors contributed to unsustainable grazing practices, i.e. development agencies promote livestock productivity increase as part of livelihood improvement but without accompanying carrying capacity assessments, also, communities capitalize their increased income from remittance and resources in livestock numbers due to mistrust in government, political and financial systems. Therefore, information from detailed spatial carrying capacity assessments coupled with ecological and indigenous knowledge would contribute to sustainable management of rangelands.

### 4.4 Limitations and uncertainties

However, several methodological uncertainties in assessing the carrying capacity have to be considered. Grazing area demarcations are a challenging issue given the mobility of livestock in transhumance systems and the connected social processes [102]. The presented approach included field information and survey data in connection with environmental parameters to define grazing area borders, but situation dependent crossing of respective borders by livestock and herders is most likely, particularly when resources become scarce at the end of summer. Therefore, strong contrasts in utilization intensity between the lower parts of the grazing areas in the North, such as Manjulak (2) and Alisu (3), can partly be considered as model artifact and shifts of utilization intensities have to be expected. Similarly, a border based approach does not provide information at higher spatial resolution, e.g. field observed higher utilization levels in the vicinity of pasture camps or main livestock paths cannot be reproduced with the presented model. To resolve respective issues, future approaches would benefit from increased data on livestock mobility, e.g. by systematically recording trajectories with GPS sensors [103]. This could provide the basis for weighting livestock utilization to increase the spatial resolution of the model. Furthermore, data on snow depth instead of cover, which may be generated by a radar-based approach in the future [104], would allow for an improved assessment of accessible pasture areas in winter. However, data required for such an approach would also imply substantially increased temporal, financial and human resources and the presented study provides livestock density figures on much higher spatial resolution compared to other approaches, which are usually based on districts, provinces, countries or 10 km wide pixel aggregations [10,16,17,105]. Finally, although variations exist along borders, grazing area demarcations are still a main governing factor for stocking rates in this system where informal use rights are a central aspect of livestock grazing [12]. Concentration effects of livestock on water points or in the vicinity are also actively minimized by herders that accompany most livestock in the study region and they aim for more uniform grazing within their pastures.

The various scenarios also show that the assessment results are sensitive to several modeling parameters, leading to a change of 20 percentage points in the area affected by unsustainable utilization in both alternative calculations. One reason for this is that the required energy amounts of animals vary significantly and that there are multiple sources for variation [39]. This also explains partly contradictory results on the effects of altitude and temperature on livestock metabolism [[40], [41], [42],^45^]. However, although some variations are to be expected, comparisons to existing research also show that the resulting dry matter intake figures are well within the range of existing results [43,52,106,107]. Comparing grazing potential numbers using averaged energy requirements and forage availability of the vegetation communities also supports the robustness of the assessment. This is illustrated by the example of dwarf-shrub steppes, the largest vegetation community, with a grazing potential per ha of 0.10 for yak, 0.34 for sheep, and 0.38 for goats, which is very similar to results of 0.12, 0.37 and 0.42 as found in a similar habitat by Vanselow (2011) [44].

The presented assessment considered forage quality based on existing samples of vegetation communities. Therefore, changes in species composition or related differences in the variable regions are not considered in this approach. This means that studying the overgrazing definition of e.g. Mysterud (2006) [108], i.e. the inability of forage species to survive due to herbivory pressure, would require additional species monitoring, an approach that is conducted in the research area by regular plant species surveys [14]. Additionally, repeated, stratified field sampling and derivation of total digestible nutrients for different parts of the research area would further support the reliability of the carrying capacity assessment.

### 4.5 Biomass model

The biomass model strongly increases the resolution and reliability of the assessment by the integration of extensive field data for model creation and evaluation. Although performance metrics indicate some uncertainties and low vegetation cover is challenging for remote sensing based assessments, biomass model errors show reasonable performance compared to existing research in rangeland ecosystems [78,[109], [110], [111], [112]]. Furthermore, independent field data for the year 2018 showed that the spatial and temporal transferability of the model was good, with very similar performance measures compared to the spatial cross-validation results of the 2020 model. However, the increase of unsustainable utilization areas in the conservative scenario shows that further enhancements in remote sensing of drylands would greatly improve ecological sustainability assessments.

The biomass productivity assessment must be considered as a major limitation due to the low number of remaining exclosure plots. Nevertheless, a comparison of our modeled above ground productivity values averaged over vegetation communities shows good agreement with extensive field studies in the region. This is shown by averaged alpine grassland productivity values ranging from 787 kg/ha to 1005 kg/ha in our assessment compared to field sampled values of 860 kg/ha to 980 kg/ha [113], riparian grassland productivity of 1663 kg/ha to 1866 kg/ha in our model in comparison to exclosure values of 1300 kg/ha to 1800 kg/ha [114], and averaged dwarf-shrub steppe productivities from 357 kg/ha to 556 kg/ha in our analysis compared to annual productivity of 100–500 kg/ha from field experiments in different shrub steppe types [66]. Respective results greatly increase confidence in the presented methodology, but a considerable variation is present in the literature. Future research would greatly benefit from the establishment of a network of permanent productivity measurement plots, which are distributed over a wide range of communities with different utilization levels.

As most existing studies on spatial biomass productivity used the MODIS Net Primary Production product MOD17A3 [15], a comparison of our results to respective values is of particular interest. The product is limited to regions with high vegetation cover and larger, coherent vegetated zones in our study, such as alpine grasslands, which excludes most parts of the research area. However, converting the product to biomass in kg/DM following the methodology of Oliva et al. [10] shows that the MODIS data underestimates above ground productivity by a factor of 2.7 on average, which would lead to substantially underestimated forage availability values with MODIS data and an amount that would not be able to support the field counted livestock population. This finding is supported by existing research stating a pronounced underestimation of the MODIS product, particularly in arid regions [[115], [116], [117]] and higher errors of the approach compared to our results [118]. Finally, reports of overestimation of the MODIS data exist [119]. The main easons for respective uncertainties may be biome and climate related variations of the PAR conversion efficiency, which is a key parameter to compute productivity with this approach, and uncertainties in the derivation of the leaf area index in this sparsely covered area [120]. Obtaining reliable climate data needed for such an algorithm in the research area is particularly challenging given the extremely sparse station network for validation [46]. Given the strong agreement of our productivity figures with external field data, as discussed in the previous paragraph, this indicates that the MODIS productivity product is not suitable for carrying capacity assessments in respective regions not only due to the exclusion of most steppe communities, but also due to a strong negative productivity bias in the region, a result contrary to findings of Oliva et al. [10] in Patagonia. These results show that biomass models and productivity assessments, which are adapted and validated for the specific area of interest, are an important prerequisite to assessing the grazing capacity in cold dryland regions.

## 5 Conclusion

This study provided an in-depth methodology to assess the carrying capacity in the high altitude rangelands of Central Asia. Thereby, we aimed to include all major aspects and respective spatiotemporal variations with sufficient resolution to create a sophisticated foundation for conservation and environmental management decisions. Several new methods, such as the application of snow maps for livestock accessibility or the combination of forage quality and forage quantity, were integrated to evaluate the carrying capacity at higher detail. Validation of partial results, such as biomass amounts, productivity and degradation patterns showed good agreement and support the robustness of the carrying capacity assessment. However, limited field based biomass productivity values together with remote sensing based limitations in arid regions still leads to notable uncertainties of the approach. Hence, future research on respective issues is recommended in these environments. We believe that the presented assessment study provides a valuable example for deriving the carrying capacity for cold drylands at a new level of detail and allows fact-based decisions in applied ecological management.

## Funding information

UNDP GEF grant AA/Pj/PIMS: 00076820/0088001/5038. Fondation Segré grant "Transboundary Conservation of Mountain Monarchs in Afghanistan and Pakistan”. European Union project “Improving participatory management and efficiency of rangeland and watershed focusing on Wakhan, Yakawlang, Kahmard and Saighan Districts (Contract ACA/2018/399–742)”.

## Data availability statement

Data will be made available on request. Requests to access the datasets should be directed to H.Z. and S.P.F (spoyafaryabi@wcs.org). Remote sensing datasets are available from the respective sources in the reference list.

## Declarations

Review and/or approval by an ethics committee was not needed for this study because no personal or patient data was involved in the analysis.

## CRediT authorship contribution statement

**Harald Zandler:** Writing – review & editing, Writing – original draft, Visualization, Validation, Supervision, Software, Methodology, Formal analysis, Data curation, Conceptualization. **Kim André Vanselow:** Writing – review & editing, Methodology, Conceptualization. **Sorosh Poya Faryabi:** Writing – review & editing, Project administration, Funding acquisition, Data curation, Conceptualization. **Ali Madad Rajabi:** Resources, Investigation, Data curation. **Stephane Ostrowski:** Writing – review & editing, Supervision, Resources, Project administration, Methodology, Funding acquisition, Conceptualization.

## Declaration of competing interest

The authors declare that they have no known competing financial interests or personal relationships that could have appeared to influence the work reported in this paper.

## References

[1] Louhaichi M., Gamoun M., Gouhis F. (2021). Benefits of short-duration, high-stocking rate opportunistic grazing on arid rangelands during favorable conditions. Front. Ecol. Evol..

[2] Tietjen B., Jeltsch F. (2007). Semi-arid grazing systems and climate change: a survey of present modelling potential and future needs: grazing systems and climate change. J. Appl. Ecol..

[3] Creamer M., Horback K. (2021). Researching human-cattle interaction on rangelands: challenges and potential solutions. Animals.

[4] Gaitán J.J. (2018). Aridity and overgrazing have convergent effects on ecosystem structure and functioning in patagonian rangelands: aridity and grazing effects on rangelands. Land Degrad. Dev..

[5] Zhang Y. (2015). Effects of grazing and climate warming on plant diversity, productivity and living state in the alpine rangelands and cultivated grasslands of the Qinghai-Tibetan Plateau. Rangel. J..

[6] Getabalew M., Alemneh T. (2019).

[7] Li M. (2021). Climate variability rather than livestock grazing dominates changes in alpine grassland productivity across tibet. Front. Ecol. Evol..

[8] Jansen V.S., Traynor A.C.E., Karl J.W., Lepak N., Sprinkle J. (2022). Monitoring grazing use: strategies for leveraging technology and adapting to variability. Rangelands.

[9] Vanselow K.A., Zandler H., Samimi C., Mueller L., Sychev V.G., Dronin N.M., Eulenstein F. (2021). Exploring and Optimizing Agricultural Landscapes.

[10] Oliva G., Paredes P., Ferrante D., Cepeda C., Rabinovich J. (2019). Remotely sensed primary productivity shows that domestic and native herbivores combined are overgrazing Patagonia. J. Appl. Ecol..

[11] Jansen V.S., Kolden C.A., Schmalz H.J., Karl J.W., Taylor R.V. (2021). Using satellite-based vegetation data for short-term grazing monitoring to inform adaptive management. Rangel. Ecol. Manag..

[12] Vanselow K.A., Kraudzun T., Samimi C. (2012). Grazing practices and pasture tenure in the eastern Pamirs. Mt. Res. Dev..

[13] Reeves M.C. (2015). Global view of remote sensing of rangelands: evolution, applications, future pathways. Land resources monitoring, modeling, and mapping with remote sensing.

[14] Zandler H., Faryabi S.P., Ostrowski S. (2022). Contributions to satellite-based land cover classiﬁcation, vegetation quantiﬁcation and grassland monitoring in central asian highlands using sentinel-2 and MODIS data. Front. Environ. Sci..

[15] Running S., Zhao M. (2019).

[16] Cheng D. (2017). The rangeland livestock carrying capacity and stocking rate in the kailash sacred landscape in China. Journal of Resources and Ecology.

[17] de Leeuw J. (2019). Application of the MODIS MOD 17 Net Primary Production product in grassland carrying capacity assessment. Int. J. Appl. Earth Obs. Geoinf..

[18] Umuhoza J. (2021). The analysis of grassland carrying capacity and its impact factors in typical mountain areas in Central Asia—a case of Kyrgyzstan and Tajikistan. Ecol. Indicat..

[19] Fenetahun Y., Yuan Y., Xu X.-W., Wang Y.-D. (2022). Borana rangeland of southern Ethiopia: estimating biomass production and carrying capacity using field and remote sensing data. Plant Diversity S2468265922000361.

[20] Qin P. (2021). Estimation of grassland carrying capacity by applying high spatiotemporal remote sensing techniques in zhenglan banner, inner Mongolia, China. Sustainability.

[21] Yu L., Zhou L., Liu W., Zhou H.-K. (2010). Using remote sensing and GIS technologies to estimate grass yield and livestock carrying capacity of alpine grasslands in golog prefecture, China. Pedosphere.

[22] Adjorlolo C., Botha J.O., Mhangara P., Mutanga O., Odindi J., Neale C.M.U., Maltese A. (2014). Integrating Remote Sensing and Conventional Grazing/browsing Models for Modelling Carrying Capacity in Southern African Rangelands.

[23] Zumo I.M., Hashim M., Hassan N.D. (2021). Modelling grass land carrying capacity from satellite remote sensing. IOP Conf. Ser. Earth Environ. Sci..

[24] Meshesha D.T., Moahmmed M., Yosuf D. (2019). Estimating carrying capacity and stocking rates of rangelands in Harshin District, Eastern Somali Region, Ethiopia. Ecol. Evol..

[25] Doan T., Guo X. (2019). Understanding Bison carrying capacity estimation in northern great plains using remote sensing and GIS. Can. J. Rem. Sens..

[26] Vanselow K.A., Zandler H., Samimi C. (2021). Time series analysis of land cover change in dry mountains: insights from the Tajik Pamirs. Rem. Sens..

[27] Unger-Shayesteh K. (2013). What do we know about past changes in the water cycle of Central Asian headwaters? A review. Global Planet. Change.

[28] Viviroli D., Durr H.H., Messerli B., Meybeck M., Weingartner R. (2007). Mountains of the world, water towers for humanity: typology, mapping, and global significance. Water Resour. Res..

[29] Zandler H., Morche T., Samimi C. (2017). Wind and solar power as possible energy laternatives in peripheral high mountains? Insights from the Eastern Pamirs of Tajikistan. SDMT.

[30] Zandler H., Senftl T., Vanselow K.A. (2020). Reanalysis datasets outperform other gridded climate products in vegetation change analysis in peripheral conservation areas of Central Asia. Sci. Rep..

[31] Ostrowski S., Strindberg S. (2015). Analysis of group sizes of wild ungulates recorded by community rangers in the Wakhan District, Afghanistan (2008– 2014). Wildlife Conservation Society Afghanistan Program.

[32] Smallwood P.D., Shank C.C., Lookingbill T.R., Smallwood P.D. (2019).

[33] Soelberg J., Jäger A.K. (2016). Comparative ethnobotany of the Wakhi agropastoralist and the Kyrgyz nomads of Afghanistan. J. Ethnobiol. Ethnomed..

[34] Nasa J.P.L. (2013).

[35] GADM (2018). https://gadm.org/license.html.

[36] Cabezas-Garcia E.H., Lowe D., Lively F. (2021). Energy requirements of beef cattle: current energy systems and factors influencing energy requirements for maintenance. Animals.

[37] Dong Q.M., Zhao X.Q., Ma Y.S., Xu S.X., Li Q.Y. (2006). Live-weight gain, apparent digestibility, and economic benefits of yaks fed different diets during winter on the Tibetan plateau. Livest. Sci..

[38] Linghao H., Shujie L., Shatuo C., Jianlin H., Richard C., Hanotte O., McVeigh C., Rege J.E.O. (2002). *Yak production in Central Asian highlands. Proceedings of the Third International Congress on Yak held in Lhasa, P.R. China, 4–9 September 2000* 572 (ILRI.

[39] Nagy K.A., Girard I.A., Brown T.K. (1999). Energetics of free-ranging mammals, reptiles, and birds. Annu. Rev. Nutr..

[40] Bueno M.D.I. (2018).

[41] Clarke A., Rothery P., Isaac N.J.B. (2010). Scaling of basal metabolic rate with body mass and temperature in mammals. J. Anim. Ecol..

[42] Degen A.A., Young B.A. (2002). Effect of air temperature and energy intake on body mass, body composition and energy requirements in sheep. J. Agric. Sci..

[43] National Research Council (1981).

[44] Vanselow K.A. (2011).

[45] Han X.-T., Xie A.-Y., Bi X.-C., Liu S.-J., Hu L.-H. (2003). Effects of altitude, ambient temperature and solar radiation on fasting heat production in yellow cattle (*Bos taurus*). Br. J. Nutr..

[46] Zandler H., Haag I., Samimi C. (2019). Evaluation needs and temporal performance differences of gridded precipitation products in peripheral mountain regions. Sci. Rep..

[47] Ostrowski S., Rajabi A.M., Rooyesh H. (2021).

[48] Aggarwal R.A.K. (2007).

[49] Lensch J. (1999). World Animal Review.

[50] Muhammad F. (2006). Relationship of body weight with linear body measurements in goats. J. Anim. Vet. Adv..

[51] Tolobekova A. (2019).

[52] Wiener G., Jianlin H., Ruijun L. (2003).

[53] Xue B., Zhao X.Q., Zhang Y.S. (2005). Seasonal changes in weight and body composition of yak grazing on alpine-meadow grassland in the Qinghai-Tibetan plateau of China 1. J. Anim. Sci..

[54] QGIS Development Team (2022).

[55] Hall D.K., Riggs G.A. (2016).

[56] Harris R.B. (2010). Argali abundance in the Afghan Pamir using capture–recapture modeling from fecal DNA. J. Wildl. Manag..

[57] Rooyesh H. (2021).

[58] Ahmed T., Shoeb M., Chandan P., Khan A. (2016). On the status of the long-tailed marmot Marmota caudata (mammalia: rodentia: sciuridae) in kargil, ladakh (Indian trans-himalaya). J. Threat. Taxa.

[59] Qureshi B. ud D., Anwar M., Hussain I., Azhar Beg M. (2015). New record of distribution and population density of Golden Marmot (Marmota caudata) from District Neelum, AJ&K, Pakistan. Int. J. Biosci..

[60] Körtner G., Heldmaier G. (1995). Body weight cycles and energy balance in the alpine marmot (Marmota marmota). Physiol. Zool..

[61] Fedosenko A.K., Blank D.A. (2001). Capra sibirica. Mamm. Species.

[62] Fedosenko A.K., Blank D.A. (2005). Ovis ammon. Mamm. Species.

[63] Giacometti M., Bassano B., Peracino V., Ratti P. (1997). Die Konstitution des Alpensteinbockes (Capra i. ibex L.) in Abhängigkeit von Geschlecht, Alter, Herkunft und Jahreszeit in Graubünden (Schweiz) und im Parco Nazionale Gran Paradiso (Italien). Z. Jagdwiss..

[64] Moheb Z. (2022). Using double-observer surveys to monitor urial and ibex populations in the Hindu Kush of Wakhan National Park, Afghanistan. Oryx 1–7.

[65] Green S., Brazee B. (2012). Harvest efficiency in prescribed grazing. USDA - Natural Resources Conservation Service TN RANGE NO..

[66] Jusufbekov C.J. (1968).

[67] Weiss W.P., Conrad H.R., Pierre N.R.S. (1992). A theoretically-based model for predicting total digestible nutrient values of forages and concentrates. Anim. Feed Sci. Technol..

[68] Van Soest P.J. (1994).

[69] Menke K.H., Huss W. (1987).

[70] Ding L.M. (2014). Seasonal heat production and energy balance of grazing yaks on the Qinghai-Tibetan plateau. Anim. Feed Sci. Technol..

[71] Louhaichi M., Gamoun M., Hassan S., Abdallah M.A.B. (2021). Characterizing biomass yield and nutritional value of selected indigenous range species from arid Tunisia. Plants.

[72] Robles A.B., Ruiz-Mirazo J., Ramos M.E., González, Rebollar J.L., Rigueiro-Rodróguez A., McAdam J., Mosquera-Losada M.R. (2008).

[73] Stergiadis S., Allen M., Chen X., Wills D., Yan T. (2015). Prediction of metabolisable energy concentrations of fresh-cut grass using digestibility data measured with non-pregnant non-lactating cows. Br. J. Nutr..

[74] Mueller-Dombois D., Ellenberg H. (1974).

[75] ESA (2020). https://earth.esa.int/web/guest/missions/esa-operational-eo-missions/sentinel-2.

[76] (2018). ESA. Sen2Cor v2.5.5 Atmospheric Correction Software.

[77] S2 MSI ESL team (2023). Sentinel-2 L1C data quality report OMPC.CS.DQR.01.05-2023. Sentinel-2 L1C Data Quality Report.

[78] Zandler H., Brenning A., Samimi C. (2015). Quantifying dwarf shrub biomass in an arid environment: comparing empirical methods in a high dimensional setting. Rem. Sens. Environ..

[79] Schumacher P. (2016). Do red edge and texture attributes from high-resolution satellite data improve wood volume estimation in a semi-arid mountainous region?. Rem. Sens..

[80] Qi J., Chehbouni A., Huete A.R., Kerr Y.H., Sorooshian S. (1994). A modified soil adjusted vegetation index. Rem. Sens. Environ..

[81] Escadafal R., Huete A. (1991). Étude des propriétés spectrales des sols arides appliquée à l’amélioration des indices de végétation obtenus par télédétection. C. R. Acad. Sci. Paris.

[82] Rajah P., Odindi J., Mutanga O., Kiala Z. (2019). The utility of Sentinel-2 Vegetation Indices (VIs) and Sentinel-1 Synthetic Aperture Radar (SAR) for invasive alien species detection and mapping. NC.

[83] Eitel J.U.H., Long D.S., Gessler P.E., Hunt E.R., Brown D.J. (2009). Sensitivity of ground-based remote sensing estimates of wheat chlorophyll content to variation in soil reflectance. Soil Sci. Soc. Am. J..

[84] Ramoelo A. (2012). Regional estimation of savanna grass nitrogen using the red-edge band of the spaceborne RapidEye sensor. Int. J. Appl. Earth Obs. Geoinf..

[85] Ren H., Zhou G., Zhang F., Zhang X. (2012). Evaluating cellulose absorption index (CAI) for non-photosynthetic biomass estimation in the desert steppe of Inner Mongolia. Chin. Sci. Bull..

[86] Breiman L. (2001). Random forests. Mach. Learn..

[87] Raab C. (2020). Target‐oriented habitat and wildlife management: estimating forage quantity and quality of semi‐natural grasslands with Sentinel‐1 and Sentinel‐2 data. Remote Sens Ecol Conserv.

[88] Vanselow K.A., Samimi C. (2014). Predictive mapping of dwarf shrub vegetation in an arid high mountain ecosystem using remote sensing and random forests. Rem. Sens..

[89] Diesing M. (2020). https://essd.copernicus.org/preprints/essd-2020-22/.

[90] Bischl B. (2016). Machine learning in R. Journal of Machine Learning Researc.

[91] Schratz P., Becker M., Lang M., Brenning A. (2021).

[92] Schratz P., Becker M. (2021).

[93] McNaughton S.J., Milchunas D.G., Frank D.A. (1996). How can net primary productivity be measured in grazing ecosystems?. Ecology.

[94] Lauriault L.M., Schmitz L.H., Cox S.H., Duff G.C., Scholljegerdes E.J. (2022). A comparison of native grass and triticale pastures during late winter for growing cattle in semiarid, subtropical regions. Agronomy.

[95] Shawver C.J., Ippolito J.A., Brummer J.E., Ahola J.K., Rhoades R.D. (2021). Soil health changes following transition from an annual cropping to perennial management‐intensive grazing agroecosystem. Agrosyst. geosci. environ..

[96] Woods S.R., Ruyle G.B. (2015). Informal rangeland monitoring and its importance to conservation in a U.S. Ranching community. Rangel. Ecol. Manag..

[97] Holechek J.L. (1988). An approach for setting the stocking rate. Rangelands.

[98] Shikui D., Ruijun L., Zizhi H., Luming D., Meiyong X. (2003). Influence of grazing intensity on performance of perennial grass mixtures in the alpine region of the Tibetan Plateau. New Zealand Journal of Agricultural Research.

[99] Yu L., Zhou L., Liu W., Zhou H.-K. (2010). Using remote sensing and GIS technologies to estimate grass yield and livestock carrying capacity of alpine grasslands in golog prefecture, China. Pedosphere.

[100] Zandler H. (2018).

[101] Oba G., Kotile D.G. (2001). Assessments of landscape level degradation in southern Ethiopia: pastoralists versus ecologists. Land Degrad. Dev..

[102] Turner M.D., McPeak J.G., Ayantunde A. (2014). The role of livestock mobility in the livelihood strategies of rural peoples in semi-arid west africa. Hum. Ecol..

[103] Feldt T., Schlecht E. (2016). Analysis of GPS trajectories to assess spatio-temporal differences in grazing patterns and land use preferences of domestic livestock in southwestern Madagascar. Pastoralism.

[104] Lievens H. (2019). Snow depth variability in the Northern Hemisphere mountains observed from space. Nat. Commun..

[105] Piipponen J. (2022). Global trends in grassland carrying capacity and relative stocking density of livestock. Global Change Biol..

[106] Boke-Olén N., Lehsten V., Abdi A.M., Ardö J., Khatir A.A. (2018). Estimating grazing potentials in Sudan using daily carbon allocation in dynamic vegetation model. Rangel. Ecol. Manag..

[107] National Research Council (1975).

[108] Mysterud A. (2006). The concept of overgrazing and its role in management of large herbivores. Wildl. Biol..

[109] Chen Y., Guerschman J., Shendryk Y., Henry D., Harrison M.T. (2021). Estimating pasture biomass using sentinel-2 imagery and machine learning. Rem. Sens..

[110] Diouf A. (2015). Fodder biomass monitoring in sahelian rangelands using phenological metrics from FAPAR time series. Rem. Sens..

[111] Jansen V.S., Kolden C., Schmalz H. (2018). The development of near real-time biomass and cover estimates for adaptive rangeland management using landsat 7 and landsat 8 surface reflectance products. Rem. Sens..

[112] Lang M., Mahyou H., Tychon B. (2021). Estimation of rangeland production in the arid oriental region (Morocco) combining remote sensing vegetation and rainfall indices: challenges and lessons learned. Rem. Sens..

[113] Walter H., Breckle S.W. (1986).

[114] Ladygina G.M., Litvinova N.P. (1974). Productivity of selected meadow-communities in the eastern Pamirs. Problemy Botaniki.

[115] Pan N., Wang S., Wei F., Shen M., Fu B. (2021). Inconsistent changes in NPP and LAI determined from the parabolic LAI versus NPP relationship. Ecol. Indicat..

[116] Roberts G., Wooster M., Xu W., He J. (2018). Fire activity and fuel consumption dynamics in sub-saharan africa. Rem. Sens..

[117] Sjöström M. (2013). Evaluation of MODIS gross primary productivity for Africa using eddy covariance data. Rem. Sens. Environ..

[118] John R. (2013). Modelling gross primary production in semi-arid Inner Mongolia using MODIS imagery and eddy covariance data. Int. J. Rem. Sens..

[119] Fensholt R., Sandholt I., Rasmussen M.S. (2004). Evaluation of MODIS LAI, fAPAR and the relation between fAPAR and NDVI in a semi-arid environment using in situ measurements. Rem. Sens. Environ..

[120] Running S., Zhao M. (2019).

